# Prognostic Influence of Residual Tumor-Infiltrating Lymphocyte Subtype After Neoadjuvant Chemotherapy in Triple-Negative Breast Cancer

**DOI:** 10.3389/fonc.2021.636716

**Published:** 2021-11-09

**Authors:** Jesse Lopes da Silva, Lucas Zanetti de Albuquerque, Fabiana Resende Rodrigues, Guilherme Gomes de Mesquita, Priscila Valverde Fernandes, Luiz Claudio Santos Thuler, Andreia Cristina de Melo

**Affiliations:** ^1^ Division of Clinical Research and Technological Development, Brazilian National Cancer Institute, Rio de Janeiro, Brazil; ^2^ Division of Pathology, Brazilian National Cancer Institute, Rio de Janeiro, Brazil

**Keywords:** triple-negative breast cancer, tumor-infiltrating lymphocyte, tumor microenvironment, neoadjuvant chemotherapy, biomarkers

## Abstract

**Objective:**

This study aimed to examine the prevalence and prognostic role of tumor microenvironment (TME) in triple-negative breast cancer (TNBC) after neoadjuvant chemotherapy (NACT) through immunohistochemical characterization.

**Methods:**

The internal database of the Brazilian National Cancer Institute for women diagnosed with TNBC who underwent NACT and thereafter curative surgery between January 2010 and December 2014 was queried out. Core biopsy specimens and tissue microarrays containing surgical samples of TNBC from 171 and 134 women, respectively, were assessed by immunohistochemistry for CD3, CD4, CD8, CD14, CD56, CD68, CD117, FOXP3, PD-1, PD-L1, and PD-L2. Immune cell profiles were analyzed and correlated with response and survival.

**Results:**

Mean age was 50.5 years, and most cases were clinical stage III [143 cases (83.6%)]. According to the multivariate analysis, only Ki67 and clinical stage significantly influenced the pattern of response to systemic treatment (*p* = 0.019 and *p* = 0.033, respectively). None of the pre-NACT IHC markers showed a significant association with event-free survival (EFS) or overall survival (OS). As for post-NACT markers, patients with high CD14 had significantly shorter EFS (*p* = 0.015), while patients with high CD3 (*p* = 0.025), CD4 (*p* = 0.025), CD8 (*p* = 0.030), CD14 (*p* = 0.015), FOXP3 (*p* = 0.005), high CD4/FOXP3 (*p* = 0.034), and CD8/FOXP3 (*p* = 0.008) showed longer EFS. Only high post-NACT CD4 showed significantly influenced OS (*p* = 0.038).

**Conclusion:**

The present study demonstrated that the post-NACT TIL subtype can be a determining factor in the prognosis of patients with TNBC.

## Introduction

Triple-negative breast cancer (TNBC) is defined by the lack of expression of estrogen receptor (ER), progesterone receptor (PR), and human epidermal growth factor receptor 2 (HER2) overexpression (1, 2). Accounting for about 10%–15% of breast cancer cases worldwide, this subtype amounts to over 300,000 new cases in women each year (3, 4). TNBC stands out for its aggressiveness, invasiveness, and high recurrence rate in the first 3 years after treatment, when compared to other types of breast cancer (5).

Recently, a growing trend has emerged toward treating patients with early-stage disease with neoadjuvant chemotherapy (NACT), supported by some plausible advantages: a significant increase in survival in patients with pathological complete response (pCR), the possibility of conversion to breast-conserving surgery, elimination of micrometastatic disease, as well as *in vivo* sensitivity test to chemotherapy (6, 7). Tumor microenvironment (TME) heterogeneity, represented by tumor-infiltrating lymphocytes (TILs) and other types of immune cells in different proportions, has been widely cited as one of the main reasons for different clinical outcomes, including patterns of response to NACT and survival (8).

Several studies have been published suggesting a crucial role of the TME in carcinogenesis. In this context, some results suggest that TILs are mostly found in highly proliferative tumors, such as TNBC and HER2-positive breast cancers, influencing outcomes such as pathologic response to NACT as well as recurrence and survival (9–12).

More specifically, some interleukins (IL) secreted by specific infiltrated cell subtypes, such as IL-6 and IL-8, may exert a sustained stimulatory mechanism as loop feedback between the TME and cancer cells, impairing tumor growth by an immune attack. However, depending on the subtype of TILs, this effect can be inhibitory or stimulatory for breast cancer progression. T helper (CD4+) and cytotoxic (CD8+) lymphocytes, primary effector TIL subtypes, have been positively associated with a higher response rate to chemotherapy and with better overall survival (OS).

Conversely, infiltration by FOXP3+ regulatory T (Treg) cells is critical in maintaining immune tolerance and is likely to predict a worse prognosis by the so-called immune evasion. Likewise, strengthening this evasion of immune destruction, immune checkpoint proteins such as PD-1, PD-L1, and PD-L2 are likely to play an important role, not only in immunotherapy effectiveness but also in the anti-tumor effects of conventional anti-tumor drugs (13, 14). The CD56 highly expressed, commonly related to tumor-infiltrating natural killer (NK) cells (CD56+ NK-TILs), has shown discrepant findings among different types of tumors. It is known that NK cells are effector lymphocytes, a component of the innate immune system, and play an immunoregulatory part by inhibiting tumor growth and spread (15).

The predictive and prognostic function of immune biomarkers in TNBC remains unclear. This study aimed to investigate the influence of TILs, as well as PD-1, PD-L1, and PD-L2 expression, in the response pattern to NACT and survival outcomes in the subset of patients with locally advanced TNBC.

## Materials and Methods

### Study Design and Ethical Considerations

This is a cohort study with retrospective data. The study was approved by the Ethics in Human Research Committee and conducted following the Good Clinical Practice guidelines.

### Patient Selection

Patients with newly diagnosed breast cancer enrolled at the Brazilian National Cancer Institute (INCA) between January 2010 and December 2014 were included if all the following criteria were met: (a) women over 18 years old; (b) confirmation of the histopathological diagnosis of TNBC by the INCA Division of Pathology (DIPAT-INCA) following the criteria of the American Society of Clinical Oncology/College of American Pathologists (ASCO/CAP) guidelines (1, 2): tumors with ER and PR score <1%, as well as HER2 score 0/1+ or 2+ with negative FISH; (c) stage IIb–IIIc by the 7th American Joint Committee on Cancer—AJCC (T3-4NanyM0; TanyN1-3M0); (d) submitted to NACT with anthracycline-taxane-based regimen (**Supplementary Box 1**) followed by curative surgery at INCA; and (e) the NACT was supplemented by complementary treatment with further chemotherapy and/or radiotherapy before surgery in some cases. On the other hand, the exclusion criteria comprised patients previously exposed to antineoplastic agents, with second primary or unresectable tumors after neoadjuvant treatment.

### Immunohistochemistry

Due to the scarcity of material, the core biopsy samples were analyzed in their whole tissue sections for all biomarkers of IHC. As for the surgical specimen samples, the tissue microarray (TMA) analysis was performed using standard procedures on 4-μm sections in the three most representative areas of greatest tumor cellularity of formalin-fixed paraffin-embedded tissue specimens, and then stained for each biomarker, taking the highest value for the purpose of final scoring. For both specimens, the tumor cell staining was compared with that of negative and positive controls.

Samples were immunostained for ER (clone EP1, Dako, prediluted), PR (clone PgR636, Dako, prediluted), HER2 (clone SP3, Cell Marque, diluted 1:500), CD3 (clone MRQ-39, Cell Marque, diluted 1:1,000), CD4 (clone SP35, Cell Marque, diluted 1:400), CD8 (clone SP 16, Cell Marque, diluted 1:1,000), CD14 (clone EPR3653, Cell Marque, diluted 1:200), CD56 (clone 123C3.D5, Cell Marque, diluted 1:800), CD68 (clone Kp-1, Cell Marque, diluted 1:1,500), FOXP3 (clone 236A/E7, Abcam, diluted 1:50), PD-1 (clone NAT105, Cell Marque, diluted 1:100), PD-L1 (clone SP142, Ventana, prediluted), and PD-L2 (clone ab200377, Abcam, diluted 1:200).

The immunostaining scores for ER, PR, and HER2 were confirmed as negative according to ASCO/CAP guidelines (16). Ki67 was assessed by nuclear staining using a mouse monoclonal antibody (SP6 clone, Cell Marque) at 1:500 dilution. Intratumoral stromal immune markers were manually counted and scored by two experienced pathologists using a double-blind method as described hereafter. The tumor cell staining was compared with that of negative controls made from counterstaining with hematoxylin and of positive controls. In core biopsy and TMA specimens of surgical samples with residual tumor, for TIL subpopulations (CD3+, CD4+, CD8+, and FOXP3+), intratumoral stromal lymphocytes were counted semi-automatically and quantified as the average absolute number of immunolabeled lymphocytes at each of three selected field at 200× magnification.

In core biopsy specimens, for PD-1 and PD-L2, the slides were scored according to the percentage of positive cells divided by the number of fields to calculate the mean value for each case, determined at 200× magnification. The PD-L1 tumor proportion score (TPS) was defined as the percentage of viable tumor cells showing partial or complete membrane staining at any intensity. PD-L1 expression on immune cell (IC) was assessed as the proportion of tumor area occupied by PD-L1-positive IC of any intensity. The PD-L1 combined positive score (CPS) refers to the ratio between PD-L1-positive cells (tumor or immune cells) and the total number of tumor cells × 100, and was grouped here in negative (<1) or positive (≥ 1) status.

For statistical analysis, the cutoff points for most biomarkers were calculated using the surv_cutpoint function of the survminer R package to divide the scores into low- and high-level groups. In this context, considering the core biopsy markers and surgical specimen, the cutoff points are stated in **Supplementary Tables 1**, **2**, respectively.

### Other Pathological and Clinical Variables

The data were collected from electronic hospital records and medical charts. The following clinical and pathological variables were retrieved: age at diagnosis, ethnicity (Caucasian or others according to Brazilian Institute of Geography and Statistics, IBGE) (17), body mass index (BMI), clinical stage (II–III), clinical T stage (cT), clinical nodal stage (cN), residual cancer burden (RCB), histological type, Elston histological grade (1: low grade; 2: moderate grade; 3: high grade), type of NACT (FAC-T or AC-T), complementary treatment, and type of surgery (radical or conservative, axillary approach).

### Statistical Analysis

The RCB score followed the standard four-level categorical variable (RCB “classes” 0, 1, 2, and 3) (18). The pCR, similarly to RCB-0, was narrowly defined as no viable residual tumor in the breast or axilla (ypT0N0). Event-free survival (EFS) was calculated from the date of diagnosis to the earliest date of disease progression, death from any cause, or discontinuation of treatment for initiation of complementary treatment due to poor response to standard NACT. OS was calculated from the date of diagnosis to death from any cause or censored if the patient was known to be alive on the last day of data collection. All continuous variables were evaluated by the Shapiro–Wilk test of normality. For the RCB outcome, logistic regression was used for each variable assessed. The Kaplan–Meier method was used to estimate EFS and OS for each variable and was compared by the log-rank test. The Cox proportional hazards model was used to calculate the crude hazard ratio (HR) for each factor. Regarding multivariate analysis, all variables with an association with response and survival outcomes at *p*-value <0.20 were included, and the Akaike Information Criterion was used to pick the most suitable model for multiple Cox analysis. A *p*-value <0.05 was considered statistically significant. The missing data were excluded from the analysis. The statistical analyses were conducted using R environment version 3.5.3.

## Results

As shown in the study profile description in **Supplementary Figure 1**, 171 women with TNBC treated with anthracycline-taxane-based NACT were included for evaluation. Detailed information regarding clinicopathological features and treatment data is highlighted in **Supplementary Table 3**. Mean age was 50.5 years (standard deviation, SD 10.7), most cases were clinical stage III [143 cases (83.6%)], and the mean BMI was 28.5 kg/m² (SD 5.8). Invasive ductal carcinoma not otherwise specified (NOS) was the most prevalent histology [160 cases (93.6%)] and more than half of the cases were histological grade 3 [115 cases (67.3%)]. Neoadjuvant treatment predominantly consisted of AC-T [117 cases (68.4%)], and the pCR rate was 21% (36 cases). The vast majority of patients underwent mastectomy [165 cases (96.5%)] and axillary dissection [145 cases (93.5%)].

Median pre-NACT scores and cutoff points for IHC biomarkers for the general population are shown in **Supplementary Table 1**. CD3 [median (IQR), cutoff point; 10 (29), 5.00] was the most predominant lymphomononuclear subpopulation, with lower values for CD14 [1 (4)] and CD117 [0 (1)], while no CD56 subpopulation was found, which made it unviable to perform the analyses of this marker for previously determined outcomes. The numbers for the ratios were as follows: CD4/FOXP3 [0.67 (2.90), 5.00], CD8/FOXP3 [0.50 (0.91), 3.27], and CD4/CD8 [1.00 (1.48), 5.50]. As for the PD1/PD-L1/PD-L2 axis immune checkpoint signaling, most of the patients presented PD-1 low [144 cases (93.5%)], PD-L1 IC <1% [138 cases (85.7%)], PD-L1 TPS low [119 cases (70.4%)], PD-L1 CPS positive [100 cases (58.8%)], and PD-L2 low [105 cases (65.2%)].

According to the data shown in **Supplementary Table 2**, a total of 134 patients had surgical samples with residual tumor and were evaluated for the IHC markers of selected TILs. CD14 was the most predominant marker [4.33 (12.67), 0.33], followed by CD4 [2.67 (8.34), 4.00] and FOXP3 [2.67 (5.37), 0.67]. The results for ratios were observed as described: CD4/FOXP3 [1.31 (2.19), 0.72], CD8/FOXP3 [0.51 (0.71), 0.52], and CD4/CD8 [2.00 (4.28), 0.47].

The association of the clinical and pathological features with response to NACT was individually assessed and summarized in **Table 1**. By multivariate analysis, only Ki67 showed a significant positive correlation with the pattern of response to systemic treatment (*p* = 0.019). On the other hand, stage III showed a significantly poorer lower rate of RCB 0/I as compared to stage II (*p* = 0.033).

**Table 1:** Correlation of clinical–pathological characteristics and expression profile of biomarkers with residual burden cancer by logistic regression through univariate analysis (*n* = 171).

Variables/biomarker	RCB 0/1	RCB 2/3	Crude *p*-value	Adjusted *p*-value
	50 (29.2%)	121 (70.8%)		
Age mean (SD)	51.0 (11.5)	50.3 (10.4)	0.849	
BMI kg/m² mean (SD)	29.2 (7.2)	28.3 (5.2)		
**Clinical stage**			**0.026**	**0.033**
**II**	**13 (26%)**	**15 (12.4%)**		
**III**	**37 (74%)**	**106 (87.6%)**		
Grade			0.547	
1	1 (2.0%)	2 (1.7%)		
2	17 (34%)	36 (29.8%)		
3	32 (64%)	83 (68.6%)		
Histological type			0.433	
Invasive ductal carcinoma NOS	48 (96%)	112 (92.6%)		
Metaplastic	2 (4%)	9 (7.4%)		
NACT regimen			0.863	
AC-T	34 (68%)	83 (68.6%)		
FAC-T	16 (32%)	38 (31.4%)		
**Ki 67 mean (SD)**	51.6 (28.8)	42.5 (30.7)	0.127	**0.019**
CD3			0.907	
High	8 (16.7%)	18 (15.9%)		
Low	40 (83.3%)	95 (84.1%)		
**CD4**			**0.046**	0.147
**High**	**37 (77.1%)**	**101 (89.4%)**		
**Low**	**11 (22.9%)**	**12 (10.6%)**		
CD8			0.581	
High	23 (47.9%)	59 (52.7%)		
Low	25 (52.1%)	53 (47.3%)		
CD14			0.825	
High	7 (14.6%)	15 (13.3%)		
Low	41 (85.4%)	98 (86.7%)		
CD68			0.571	
High	24 (50%)	62 (54.9%)		
Low	24 (50%)	51 (45.1%)		
CD117			0.437	
High	5 (10.4%)	17 (15%)		
Low	43 (89.6%)	96 (85%)		
FOXP3			0.340	
High	19 (39.6%)	54 (47.8%)		
Low	29 (60.4%)	59 (52.2%)		
PD-1			0.188	0.256
High	1 (2.2%)	9 (8.3%)		
Low	45 (97.8%)	99 (91.7%)		
PD-L1 TPS			0.238	
High	18 (36%)	32 (26.9%)		
Low	32 (64%)	87 (73.1%)		
PD-L1 IC			0.944	
High	7 (14.6%)	16 (14.2%)		
Low	41 (85.4%)	97 (85.8%)		
PD-L1 CPS			0.888	
Positive	29 (58%)	71 (59.2%)		
Negative	21 (42%)	49 (40.8%)		
PD-L2			0.121	
High	21 (43.8%)	35 (31%)		
Low	27 (56.2%)	78 (69%)		
CD4/FOXP3 ratio			0.210	
High	22 (45.8%)	64 (56.6%)		
Low	26 (54.2%)	49 (43.4%)		
CD8/FOXP3 ratio			0.841	
High	41 (85.4%)	97 (86.6%)		
Low	7 (14.6%)	15 (13.4%)		
CD4/CD8 ratio			0.540	
High	4 (8.3%)	13 (11.6%)		
Low	44 (91.7%)	99 (88.4%)		

RCB, residual cancer burden; SD, standard deviation; BMI, body mass index; NOS, not otherwise specified; NACT, neoadjuvant chemotherapy; AC-T, doxorubicin/cyclophosphamide followed by taxane; FAC-T, doxorubicin/cyclophosphamide/fluorouracil; CD3, Cluster of Differentiation 3; CD4, Cluster of Differentiation 4; CD8, Cluster of Differentiation 8; CD14, Cluster of Differentiation 14; CD56, Cluster of Differentiation 56; CD68, Cluster of Differentiation 68; CD117, Cluster of Differentiation 117; FOXP3, Forkhead Box P3; PD-1, Programmed Cell Death Protein 1; PD-L1 TPS, Programmed Death-Ligand 1 tumor proportion scores; PD-L1 IC, Programmed Death-Ligand 1 tumor-infiltrating immune cells; PD-L1 CPS, Programmed Death-Ligand 1 combined positive score; PD-L2, Programmed Death-Ligand 2.

Differences in absolute value correspond to missing data.

Statistically significant results are in bold.

**Figure 1:**
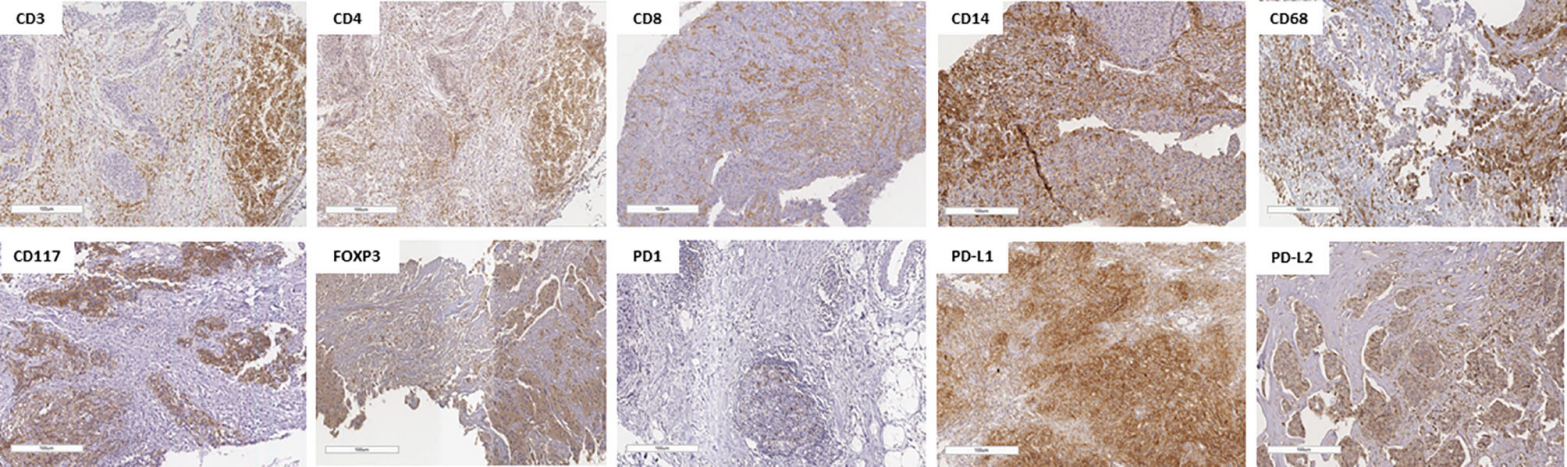
Representative pictures of lymphocyte infiltration in triple-negative breast cancer core biopsy showing immunohistochemical staining of high CD3+, CD4+, CD8+, CD14+, CD68+, CD117+, FOXP3+, PD-1+, PD-L1+, and PD-L2+. The specimens are imaged at ×20 magnification with a 100-µm scale on each one.

The median follow-up time was 62.5 months (95% confidence interval, 95% CI 60.2–67.9). As highlighted in **Supplementary Tables 4**, **5**, amid the IHC markers assessed in the core biopsy, none of them showed a significant association with the outcomes of EFS and OS. **Figure 1** shows representative images of cases with high expression of IHC markers in core biopsies.

The analysis of post-NACT clinical and pathological features in patients with residual tumors is described in **Tables 2**, **3**. By univariate analysis, patients with high CD14 had significantly shorter EFS (*p* = 0.042) than those with low expression of CD14. Conversely, high expression of CD3 (*p* = 0.007) and CD8 (*p* = 0.013), as well as high CD4/FOXP3 (*p* = 0.034) and CD8/FOXP3 (*p* = 0.008) ratios, had significantly longer EFS. The Cox model chosen for multivariate analysis was composed of six variables, five of which showed significant association for EFS: CD3 (*p* = 0.025), CD4 (*p* = 0.025), CD8 (*p* = 0.030), CD14 (*p* = 0.015), and FOXP3 (*p* = 0.005) (**Table 2**).

**Table 2:** Post-NACT clinicopathological features and crude and adjusted hazards ratios for event-free survival (EFS) estimated by univariate analysis and multivariate analysis.

Post-NACT clinicopathological features	Univariate analysis		*p*-value	Multivariate analysis		*p*-value
	HR	95% CI		HR	95% CI	
Clinical stage (III versus II)	1.91	(0.88–4.16)	0.103	1.76	(0.75–4.14)	0.194
**CD3 (high versus low)**	**0.50**	**(0.30–0.82)**	**0.007**	**0.58**	**(0.30–1.10)**	**0.025**
CD4 (high versus low)	0.69	(0.42–1.13)	0.138	**0.52**	**(0.30–0.92)**	**0.025**
**CD8 (high versus low)**	**0.41**	**(0.20–0.83)**	**0.013**	**0.37**	**(0.15–0.91)**	**0.030**
**CD14 (high versus low)**	**2.58**	**(1.03–6.45)**	**0.042**	**3.66**	**(1.29–10.41)**	**0.015**
FOXP3 (high versus low)	1.53	(0.83–2.81)	0.170	**2.78**	**(1.35–5.75)**	**0.005**
**CD4/FOXP3 ratio (high versus low)**	**0.59**	**(0.36–0.96)**	**0.034**			
**CD8/FOXP3 ratio (high versus low)**	**0.51**	**(0.31–0.84)**	**0.008**			
CD4/CD8 ratio (high versus low)	2.31	(0.83–6.41)	0.107			

CD3, Cluster of Differentiation 3; CD4, Cluster of Differentiation 4; CD8, Cluster of Differentiation 8; CD14, Cluster of Differentiation 14; CD68, Cluster of Differentiation 68; CD117, Cluster of Differentiation 117; FOXP3, Forkhead Box P3. Significant p-values are in bold.

**Table 3:** Post-NACT clinicopathological features and crude and adjusted hazards ratios for overall survival (OS) estimated by univariate analysis and multivariate analysis.

Post-NACT clinicopathological features	Univariate analysis		*p*-value	Multivariate analysis		*p*-value
	HR	95% CI		HR	95% CI	
Clinical stage (III versus II)	1.75	(0.80–3.83)	0.159	1.53	(0.65–3.60)	0.330
**CD3 (high versus low)**	**0.55**	**(0.32–0.93)**	**0.025**	0.77	(0.38–1.52)	0.449
CD4 (high versus low)	0.68	(0.41–1.14)	0.145	**0.52**	**(0.28–0.96)**	**0.038**
**CD8 (high versus low)**	**0.36**	**(0.16–0.79)**	**0.011**	0.40	(0.15–1.02)	0.055
CD14 (high versus low)	2.73	(0.99–7.57)	0.053	2.75	(0.95–7.98)	0.062
FOXP3 (high versus low)	1.63	(0.87–3.07)	0.130	2.05	(0.99–4.23)	0.051
**CD4/FOXP3 ratio (high versus low)**	**0.56**	**(0.34–0.93)**	**0.025**			
**CD8/FOXP3 ratio (high versus low)**	**0.56**	**(0.34–0.93)**	**0.026**			
CD4/CD8 ratio (high versus low)	1.96	(0.70–5.46)	0.199			

CD3, Cluster of Differentiation 3; CD4, Cluster of Differentiation 4; CD8, Cluster of Differentiation 8; CD14, Cluster of Differentiation 14; CD68, Cluster of Differentiation 68; CD117, Cluster of Differentiation 117; FOXP3, Forkhead Box P3. Significant p-values are in bold.

**Figure 2:**
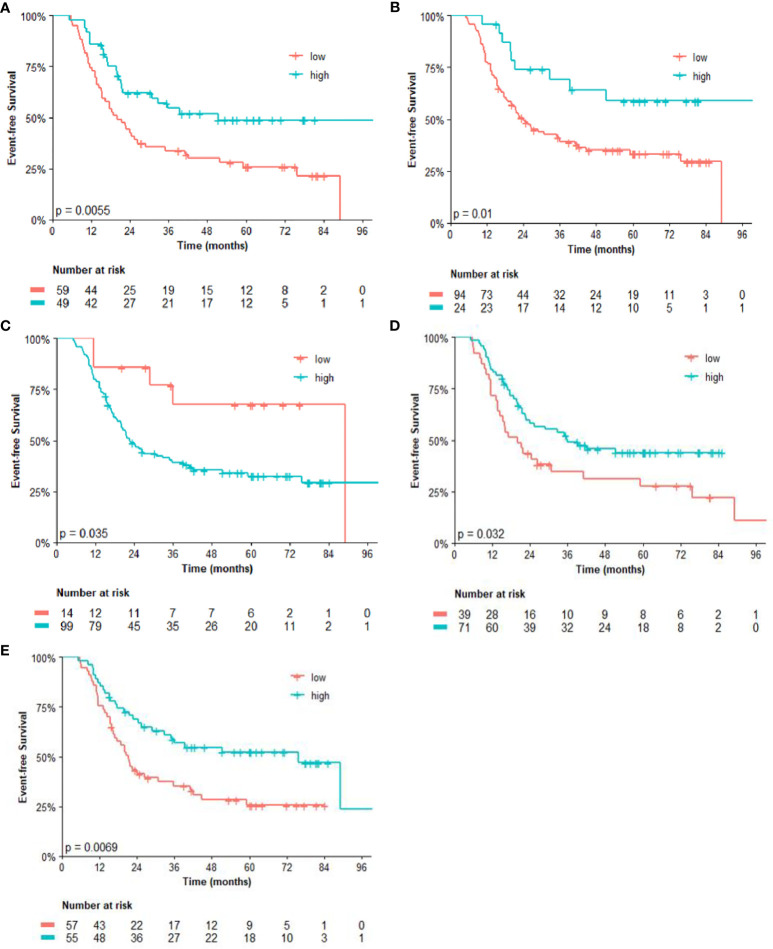
Event-free survival (EFS) by **(A)** CD3; **(B)** CD8; **(C)** CD14; **(D)** CD4/FOXP3 ratio; and **(E)** CD8/FOXP3 ratio. Regarding the immunohistochemistry markers and the ratios, Kaplan–Meier curves for EFS were stratified according to the cutoff for prognostic evaluation and divided into low versus high subgroup for each variable subsets. The red solid line indicates patients with low values and the blue solid line indicates patients with high values. Tick marks indicate censored data.

**Figure 3:**
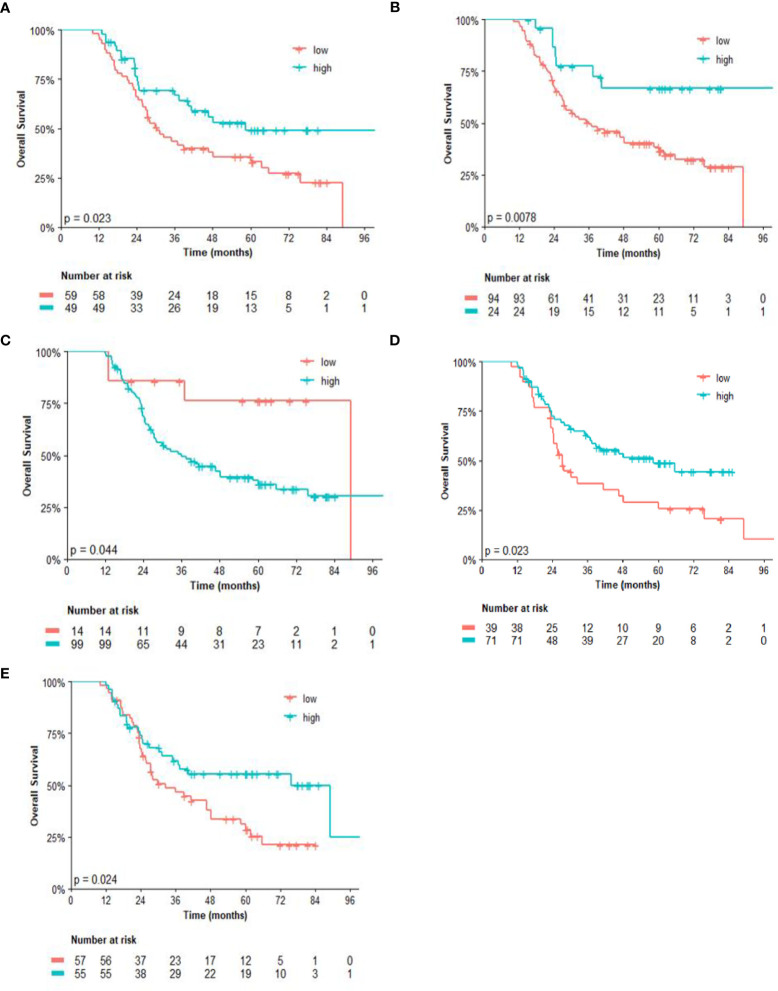
Overall survival (OS) by **(A)** CD3; **(B)** CD8; **(C)** CD14; **(D)** CD4/FOXP3 ratio; and **(E)** CD8/FOXP3 ratio. Regarding the immunohistochemistry markers and the ratios, Kaplan–Meier curves for EFS were stratified according to the cutoff for prognostic evaluation and divided into low versus high subgroup for each variable subsets. The red solid line indicates patients with low values and the blue solid line indicates patients with high values. Tick marks indicate censored data.

Regarding the univariate analysis of post-NACT clinical and pathological features for OS, patients with high CD3 (*p* = 0.025) and CD8 (*p* = 0.011), as well as high CD4/FOXP3 (*p* = 0.025) and CD8/FOXP3 (*p* = 0.026) ratios had significantly greater survival than those with low expression. Herein, of the six variables that constituted the Cox model chosen for multivariate analysis of OS, only high CD4 (*p* = 0.038) was significantly associated with lower risk of death (**Table 3**). **Figures 2**, **3** show the Kaplan–Meier curves for EFS and OS, respectively, according to the evaluated post-NACT variables.

## Discussion

In the TNBC subset, the influence of the TME in cancer cell proliferation, as well as in response to anti-cancer drugs, has been increasingly recognized over time. Furthermore, a growing body of evidence has pointed out that lymphomononuclear cell subtypes present in the TME are crucial driving factors of tumor progression and invasion. This is one of the few series to perform a thorough evaluation of TIL subtype in post-NACT residual tumors of women with TNBC. The main results suggested that the high expression of some markers in this subset might influence recurrence and death events.

The pCR (RCB-0) rate of 21.1% is similar to previous results from other published TBNC series using anthracycline and taxane-based NACT but can be considered a modest number when compared to data from treatment regimens that included new strategies such as dense-dose regimens, PARP inhibitors, immunotherapy, and antiangiogenic or platinum agents (19, 20). Amidst the IHC markers and clinicopathological features evaluated, only the clinical stage and Ki67 had a significant correlation with the pattern of response to NACT (*p* = 0.033 and 0.019, respectively) in the multivariate analysis. A phase II study conducted by Wang et al. (21) that enrolled 280 patients with stage II–III TNBC treated with neoadjuvant weekly paclitaxel and carboplatin showed that categorical and linear Ki-67 were independently correlated with pCR (*p* < 0.001). Likewise, a meta-analysis performed by Tao et al. (22) included 36 studies involving 6,793 breast cancer patients, suggesting that pretherapeutic Ki-67 LI is associated with pCR in breast cancer patients undergoing NACT (*p* < 0.001). The modified Neo-Bioscore score, validated by Mittendorf et al. (23) in a cohort that evaluated 2,377 patients with breast cancer treated with NACT, suggests that IHC markers and response to NACT be incorporated into the AJCC staging system. In the study conducted by Jamiyan et al. (24) with patients with TNBC, high intratumoral TILs were found to be more expressed in patients with stage III over stage I/II (*p* = 0.006). The association of high CD4 expression with a higher rate of pCR in TNBC cases (*p* = 0.003) was observed in the cohort performed by García-Martínez et al. (25).

The immune IHC markers assessed in core biopsy in the current study did not show a significant association with survival outcomes. Conversely, a meta-analysis performed by Gao et al. (26) with 37 studies involving patients with TNBC showed that the upregulation of TILs predicted better disease-free survival (DFS) and OS, with pooled HRs of 0.66 (95% CI, 0.57–0.76) and 0.58 (95% CI, 0.48–0.71), respectively, for TIL level (high versus low). Specifically, the CD4+ TIL subgroup (high versus low) showed a better OS (HR 0.49, 95% CI 0.32–0.76) and DFS (HR 0.54, 95% CI 0.36–0.80), and the CD8 + TIL subgroup (high versus low) showed a better DFS only (HR 0.55, 95% CI 0.38–0.81). The FOXP3 + TIL subgroup (high versus low) also showed only better DFS (HR 0.50, 95% CI 0.33–0.75), with no statistical association with OS (HR 1.28, 95% CI 0.24–6.88). In a cohort of 150 breast cancer patients performed by Rathore et al. (27), the intratumoral high CD4+ count (OR = 3.85, 95% CI = 3.28–16.71, *p* < 0.001), CD3+ (OR = 2.70, 95% CI = 1.76–8.30, *p* = 0.001), and CD8+ (OR = 2.58, 95% CI = 1.55–5.86, *p* = 0.001) showed better survival when compared to their respective low counterparts.

In another cohort with 175 infiltrating ductal carcinomas of breast, although CD56+ NK-TILs are highly expressed in 48.6% of cases, Rathore et al. (28) suggested that this marker alone may not be sufficient for predicting the survival outcomes. To explore the tumor-associated macrophages (TAMs), Wang et al. (29)evaluated the expression of CD68+ TILs in 48 samples of TNBC, showing upregulation in 71.4% of cases. Patients with high infiltration of CD68 had higher expression of inflammatory cytokines interleukin 6 (IL-6) and chemokine (C-C motif) ligand 5 (CCL-5) and lower survival rates compared to the low-infiltration group. As a marker related to TAMs, the infiltration of CD68+ cells is supposed to be positively related to tumor severity. A retrospective systematic review study conducted by Ni et al. (30) reviewed the macrophage distribution in 1,579 non-metastatic breast cancer specimens with anti-CD68 immunohistochemical staining. The data revealed that high density of CD68-TAMs was significantly related to ominous clinicopathological characteristics such as lymph node metastasis, high Ki67, poor histological grade, and hormonal receptor negativity (*p* < 0.001 for all comparisons).

Missense-specific mutations of *TP53* with loss of P53 protein function have been linked to increased expression of CD117 in some solid tumors, inhibiting cellular differentiation, proliferation, adhesion, and apoptosis (31). However, data regarding the prognostic impact of CD117 on TNBC are conflicting. Kashiwagi et al. (32) and Luo et al. (33) have suggested that CD117 protein is associated with recurrence and poor prognosis; on the other hand, other authors failed to find a significant association between CD117 and prognosis in breast cancer or TNBC (34, 35).

The immune checkpoint receptor PD-1 has a crucial role in the tumor immune evasion process. The two ligands, PD-L1 and PD-L2, have distinct expression profiles depending on the tumor types (36). Some previous studies have addressed an assessment of the influence of the PD-1/PD-L1/PD-L2 axis markers on the survival of patients with invasive breast cancer, more specifically TNBC, showing discrepant results. Mori et al. (37) demonstrated that the interaction between TILs and PD-L1 correlates with better survival outcome. In the study of Beckers et al. (38), although PD-L1 is associated with a better outcome, the results failed to show an independent prognostic role in this subset of tumors. These conflicting results could be explained by different clinical outcomes along with various chemotherapy schedules, methods of evaluation of PD-L1 expression, and definition of cutoffs. Asano et al. (14) suggested that patients with low PD-1 and PD-L1 expressions in TNBC were associated with a higher pCR rate and significantly longer DFS, and low PD-L1 expression was an independent prognostic factor.

Some studies have used immune checkpoint inhibitors as a complement to the neoadjuvant treatment strategy in patients with locally advanced TNBC. Notably, initial studies like I-SPY 2 (39) and the KEYNOTE-173 (40) trials showed that the combination of pembrolizumab, an anti-PD-1 monoclonal antibody, with NACT significantly increased the pCR rate in early-stage TNBC. An interim analysis of the phase III KEYNOTE-522 trial reported a significantly higher pCR rate (64.8% *vs*. 51.2%; *p* < 0.001) and better EFS (HR 0.63; 95% CI: 0.43–0.93) in the combination group than in the NACT alone group, regardless of PD-L1 status though. EFS was significantly higher in the pembrolizumab group after a median follow-up of 15.5 months (41). Some initial phase I/II trials suggest that the addition of durvalumab to NACT may increase the pCR to over 50% (42–44).

Some data have suggested that, in addition to the cytotoxic effect, the effectiveness of chemotherapy can also occur through the restoration of immunosurveillance inducing immunogenic cell death (45). As shown in the results of the current cohort, some subtypes of TILs present in the residual tumor such as CD3, CD8, and CD4, as well as CD4/FOXP3 and CD8/FOXP3 ratios, influenced survival outcomes. A small series with 25 consecutive patients with breast cancer reported lymphocyte activation and attraction to tumor bed in seven cases after NACT with a better prognosis in these cases (46). Ladoire et al. (47) evaluated surgical specimens from 111 patients with HER2-negative breast cancer, in which high CD8 and low FOXP3 cell infiltration after chemotherapy were significantly associated with improved RFS (*p* = 0.02) and OS (*p* = 0.002). The study conducted by Dieci et al. (48), which evaluated TILs in patients with non-pCR TNBC after NACT, suggested that the treatment could convert a low TIL into a high TIL tumor and that this conversion could be associated with a longer 5-year OS rate. García-Martínez et al. (25) identified a specific pattern of TILs in the post-NACT residual TNBC, marked by the high infiltration of CD3 and CD68, which presented poorer DFS. This discrepant result might partially be explained by the predominant infiltration of CD68, a TAM marker previously associated with poorer outcomes.

The finding that the upregulation of some subtypes of post-chemotherapy TILs could identify subgroups of patients with different prognosis paves the way for drug development and patient stratification, which could result in changes in the practical approach of patients in adjuvant treatment. Further data to unveil the mechanisms underlying the pattern of lymphomononuclear cells, as well as the changes after NACT, may determine the development of new immune-targeted therapies for breast cancer in this setting, mainly in TNBC.

The strengths of this study rely mainly on the in-depth analysis of TME data after NACT by presenting the characteristics of the lymphomononuclear infiltrate and the consequent impact on survival. The study population is homogeneous in that only patients with locally advanced TNBC who underwent NACT followed by primary surgery were included. Moreover, all core biopsy and surgical samples were double-checked by blinded experienced pathologists. Lastly, a thorough descriptive presentation of clinicopathological variables was performed and multivariate analyses reinforce the internal validity of the results.

The major limitation of the current study is its retrospective nature. So, some missing confounding factors may exist in the analysis. As a single-center study, some regional traits in the selected population may exist, and the results can be influenced by marked geographic differences. Intratumoral heterogeneity may have compromised some results from the TMA analysis. There were also many losses due to scarce material in the core biopsy. Also, it was not possible to perform any gene expression profile analysis with the available samples.

## Conclusion

The present study demonstrated that the post-NACT TIL subtype could be a determining factor in the prognosis of patients with TNBC. Undoubtedly, considering that NACT may be insufficient to achieve pCR and ensure long survival in some cases, the composition of TME in post-NACT residual tumors of TNBC could be explored in the future to guide the extension of adjuvant treatment as a longer maintenance approach.

## Data Availability Statement

The raw data supporting the conclusions of this article will be made available by the authors without undue reservation.

## Ethics Statement

This study involving human participants was reviewed and approved by the Ethics in Human Research Committee of the Brazilian National Cancer Institute, Rio de Janeiro, Brazil, under registration number CAAE 61675516.9.0000.5274, and conducted in accordance with Good Clinical Practice guidelines. The ethics committee waived the requirement of written informed consent for participation.

## Author Contributions

The study design was planned by JS and AM. JS, AM, and LA were involved in the data collection in medical records. FR, GM, and PF performed the pathologic studies, selected the samples for the tissue microarray, and participated in the optimization of the image analysis. JS, AM, and LT participated in the analysis and interpretation of the data. All authors contributed to the article and approved the submitted version.

## Funding

This study was supported by a grant provided by AstraZeneca Brazil as a study of investigator initiative (ESR-17-12857).

## Conflict of Interest

The authors declare that the research was conducted in the absence of any commercial or financial relationships that could be construed as a potential conflict of interest.

The sponsor approved the study design and the final manuscript.

## Publisher’s Note

All claims expressed in this article are solely those of the authors and do not necessarily represent those of their affiliated organizations, or those of the publisher, the editors and the reviewers. Any product that may be evaluated in this article, or claim that may be made by its manufacturer, is not guaranteed or endorsed by the publisher.
