# Supplementary material for: Prognostic Influence of Residual Tumor-Infiltrating Lymphocyte Subtype After Neoadjuvant Chemotherapy in Triple-Negative Breast Cancer
Source: Front Oncol. 2021 Nov 9;11:636716. doi: 10.3389/fonc.2021.636716 (PMC8630741; doi:10.3389/fonc.2021.636716)

Supplementary box 1. Neoadjuvant chemotherapy regimens

Standard NACT regimens	Dose/schedule
FAC-T	Fluorouracil 500 mg/m^2, doxorubicin 50 mg/m^2 and cyclophosphamide 500 mg/m^2, administered intravenously every 21 days for 3 cycles, followed by docetaxel 100 mg/m^2 every 21 days for 3 cycles.
AC-T	Doxorubicin 60 mg/m^2 and cyclophosphamide 600 mg/m^2, given intravenously every 21 days for 4 cycles, followed by docetaxel 100 mg/m^2 given intravenously every 21 days for 4 cycles, or followed by weekly paclitaxel 80 mg/m^2 given intravenously for 12 consecutive weeks without interval, defined here as a total of four 3-week cycles.
Complementary chemotherapy*	Dose/schedule
Cisplatin	75mg/m^2 administered every 21 days intravenously during radiotherapy.
Capecitabine	850 mg/m^2 orally twice daily for 14 days every 3 weeks concomitant with radiotherapy.

NACT: Neoadjuvant chemotherapy.
*Following the routine of the local oncology team, patients with tumors considered unresectable soon after NACT were exposed to complementary chemotherapy and/or salvage radiotherapy to achieve clinical response to enable the surgical approach.

Supplementary table 1. Pre-neoadjuvant chemotherapy immunohistochemistry markers (n = 171).

Marker	Median (IQR)	Cut-off point
CD3	10 (29)	5.00
High26 (16.1%)		
Low 135 (83.9%)		
CD4	1 (9)	0
High 138 (85.7%)		
Low 23 (14.3%)		
CD8	5 (10)	1.00
High 82 (51.2%)		
Low 78 (48.8%)		
CD14	1 (4)	5.00
High 22 (13.7%)		
Low 139 (86.3%)		
CD56	0 (0)	-
CD68	5 (9)	1.00
High 86 (53.4%)		
Low 75 (46.6%)		
CD117	0 (1)	3.00
High 22 (13.7%)		
Low 139 (86.3%)		
FOXP3	5 (39)	5.00
High 73 (45.3%)		
Low 88 (54.7%)		
PD-1	0 (0)	0
High 144 (93.5%)		
Low 10 (6.5%)		
PD-L1 TPS	0 (5)	1.00
High 50 (29.6%)		
Low119 (70.4%)		
PD-L1 IC	0 (0)	0
< 1% 138 (85.7%)		
1-5% 5 (3.1%)		
5-10% 10 (6.2%)		
> 10% 8 (5%)		
PD-L1 CPS	0 (1)	0
Positive 100 (58.8%)		
Negative 70 (41.2%)		
PD-L2	5 (49)	2.00
High 56 (34.8%)		
Low105 (65.2%)		
CD4/FOXP3 ratio	0.67 (2.90)	5.00
High86 (53.4%)		
Low75 (46.6%)		
CD8/FOXP3 ratio	0.50 (0.91)	3.27
High 138 (86.2%)		
Low22 (13.8%)		
CD4/CD8 ratio	1.00 (1.48)	5.50
High 17 (10.6%)		
Low 143 (89.4%)		

IQR: interquartile range; CD3: Cluster of Differentiation 3; CD4: Cluster of Differentiation 4; CD8: Cluster of Differentiation 8; CD14: Cluster of Differentiation 14; CD56: Cluster of Differentiation 56; CD68: Cluster of Differentiation 68; CD117: Cluster of Differentiation 117; FOXP3: Forkhead Box P3; PD-1: Programmed Cell Death Protein 1; PD-L1 TPS: Programmed Death-Ligand 1 tumor proportion scores;PD-L1 IC: Programmed Death-Ligand 1 tumor infiltrating immune cells; PD-L1 CPS: Programmed Death-Ligand 1 combined positive score; PD-L2: Programmed Death-Ligand 2.
Differences in absolute value correspond to missing data.

Supplementary table 2. Post-neoadjuvant chemotherapy immunohistochemistry markers in residual tumors (n = 134).

Marker	Median (IQR)	Cut-off point
CD3	2 (5.70)	2.50
High 49 (45.4%)		
Low 59 (54.6%)		
CD4	2.67 (8.34)	4.00
High 49 (41.9%)		
Low 68 (58.1%)		
CD8	0.67 (2)	2.67
High 24 (20.3%)		
Low 94 (79.7%)		
CD14	4.33 (12.67)	0.33
High 99 (87.6%)		
Low 14 (12.4%)		
FOXP3	2.67 (5.37)	0.67
High 90 (78.9%)		
Low 4 (21.1%)		
CD4/FOXP3 ratio	1.31 (2.19)	0.72
High 71 (64.5%)		
Low 39 (35.5%)		
CD8/FOXP3 ratio	0.51 (0.71)	0.52
High 55 (49.1%)		
Low 57 (50.9%)		
CD4/CD8 ratio	2.00 (4.28)	0.47
High 104 (90.4%)		
Low 11 (9.6%)		

IQR: interquartile range; CD3: Cluster of Differentiation 3; CD4: Cluster of Differentiation 4; CD8: Cluster of Differentiation 8; CD14: Cluster of Differentiation 14; FOXP3: Forkhead Box P3;
Differences in absolute value correspond to missing data.

Supplementary table 3. Clinical and pathological general features of eligible patients.

Features	n = 171
Age years mean (SD)	50.5 (10.7)
Race/ethnicity White	78 (45.6%)
BMI Kg/m² mean (SD)	28.5 (5.8)
Ki67 mean (SD)	45.1 (30.4)
Histological type	
Invasive ductal carcinoma NOS	160 (93.6%)
Metaplastic	11 (6.4%)
Clinical Staging	
II	28 (16.4%)
III	143 (83.6%)
Clinical T stage	
cTx	1 (0.6%)
cT2	23 (13.5%)
cT3	70 (40.9%)
cT4	77 (45%)
Clinical N stage	
N0	49 (28.7%)
N1-N3	122 (71.3%)
Histological grade	
Grade 1	3 (1.8%)
Grade 2	53 (31%)
Grade 3	115 (67.3%)
LVI	
Present	38 (26%)
Absent	108 (74%)
PI	
Present	21 (16.8%)
Absent	104 (83.2%)
NACT regimen	
AC-T	117 (68.4%)
FAC-T	54 (31.6%)
Type of surgery	
Breast-conserving surgery	6 (3.5%)
Mastectomy	165 (96.5%)
Axillary approach	
Sentinel lymph node biopsy	10 (6.5%)
Axillary lymph node dissection	145 (93.5%)
RCB	
0	36 (21.1%)
1	13 (7.6%)
2	74 (43.3%)
3	48 (28.1%)

SD: Standard deviation; BMI: Body mass index; NOS: not otherwise specified; LVI: lymphovascular invasion, PI: perivascular infiltration; NACT: neoadjuvant chemotherapy; AC-T: doxorubicin/cyclophosphamide followed by taxane; FAC-T: doxorubicin/cyclophosphamide/fluorouracil followed by taxane; RCB: residual cancer burden.

Differences in absolute value correspond to missing data.

Supplementary table 4. Pre-NACT clinicopathological features and Hazards Ratios for event-free survival (EFS) estimated by univariate analysis and multivariate analysis.

Pre-NACT clinicopathological features	Univariate analysis HR (95%CI)	p-value	Multivariate analysis HR (95%CI)	p-value
Age	0.99 (0.97 - 1.01)	0.285		
BMI	1.00 (0.96 - 1.03)	0.870		
**Stage (III versus II)**	**2.56 (1.18 - 5.57)**	**0.017**	**2.20(1.00 -4.86)**	**0.049**
CD3 (high versus low)	0.54 (0.26 - 1.13)	0.101	0.57 (0.26 - 1.29)	0.180
**CD4 (high versus low)**	**2.28 (0.99 - 5.25)**	**0.053**	2.51 (1.00 - 6.29)	0.050
CD8 (high versus low)	1.19 (0.76 - 1.87)	0.440		
CD14 (high versus low)	1.19 (0.63 - 2.25)	0.603		
CD68 (high versus low)	1.21 (0.77 - 1.89)	0.418		
CD117 (high versus low)	0.75 (0.36 - 1.57)	0.450		
FOXP3 (high versus low)	1.23 (0.78 - 1.92)	0.374		
PD-1 (high versus low)	1.41 (0.57 - 3.49)	0.462		
PD-L1 TPS (high versus low)	0.61 (0.36 - 1.03)	0.065	0.68 (0.39 - 1.17)	0.166
PD-L1 IC (high versus low)	1.48 (0.82 - 2.69)	0.197	1.63 (0.88 - 3.03)	0.118
PD-L1 CPS (positive versus negative)	0.81 (0.52 - 1.27)	0.364		
PD-L2 (high versus low)	1.14 (0.72 - 1.82)	0.575		
CD4/FOXP3 ratio (high versus low)	1.38 (0.88 - 2.18)	0.161		
CD8/FOXP3 ratio (high versus low)	1.31 (0.66 - 2.64)	0.442		
CD4/CD8 ratio (high versus low)	1.55 (0.82 - 2.93)	0.181		

CD3: Cluster of Differentiation 3; CD4: Cluster of Differentiation 4; CD8: Cluster of Differentiation 8; CD14: Cluster of Differentiation 14; CD68: Cluster of Differentiation 68; CD117: Cluster of Differentiation 117; FOXP3: Forkhead Box P3; PD-1: Programmed Cell Death Protein 1; PD-L1 TPS: Programmed Death-Ligand 1 tumor proportion scores; PD-L1 IC: Programmed Death-Ligand 1 tumor infiltrating immune cells; PD-L1 CPS: Programmed Death-Ligand 1 combined positive score; PD-L2: Programmed Death-Ligand 2. Significant p-values are in bold.

Supplementary table 5. Pre-NACT clinicopathological features and Hazards Ratios for overall survival (OS) estimated by univariate analysis and multivariate analysis.

Pre-NACT clinicopathological features	Univariate analysis HR (95%CI)	p-value	Multivariate analysis HR (95%CI)	p-value
Age	1.01 (0.97 - 1.01)	0.241		
BMI	0.99 (0.95 - 1.03)	0.585		
**Stage (III versus II)**	**2.20 (1.01 - 4.97)**	**0.047**	2.03 (0.92 - 4.49)	0.080
CD3 (high versus low)	0.65 (0.31 - 1.36)	0.252		
CD4 (high versus low)	2.09 (0.91 - 4.84)	0.084	2.25 (0.90 - 5.62)	0.083
CD8 (high versus low)	1.09 (0.68 - 1.74)	0.732		
CD14 (high versus low)	1.21 (0.62 - 2.37)	0.578		
CD68 (high versus low)	1.14 (0.71 - 1.83)	0.599		
CD117 (high versus low)	0.92 (0.44 - 1.92)	0.821		
FOXP3 (high versus low)	1.31 (0.81 - 2.11)	0.268		
PD-1 (high versus low)	1.82 (0.73 - 4.55)	0.199		
PD-L1 TPS (high versus low)	0.67 (0.39 - 1.15)	0.144	0.67 (0.38 - 1.17)	0.161
PD-L1 IC (high versus low)	1.53 (0.82 - 2.85)	0.185	1.69 (0.90 - 3.20)	0.104
PD-L1 CPS (positive versus negative)	0.81 (0.50 - 1.30)	0.381		
PD-L2 (high versus low)	1.09 (0.66 - 1.79)	0.740		
CD4/FOXP3 ratio (high versus low)	1.19 (0.74 - 1.91)	0.476		
CD8/FOXP3 ratio (high versus low)	1.10 (0.55 - 2.22)	0.791		
CD4/CD8 ratio (high versus low)	1.23 (0.61 - 2.48)	0.567		

CD3: Cluster of Differentiation 3; CD4: Cluster of Differentiation 4; CD8: Cluster of Differentiation 8; CD14: Cluster of Differentiation 14; CD68: Cluster of Differentiation 68; CD117: Cluster of Differentiation 117; FOXP3: Forkhead Box P3; PD-1: Programmed Cell Death Protein 1; PD-L1 TPS: Programmed Death-Ligand 1 tumour proportion scores; PD-L1 IC: Programmed Death-Ligand 1 tumor infiltrating immune cells; PD-L1 CPS: Programmed Death-Ligand 1 combined positive score; PD-L2: Programmed Death-Ligand 2.

Significant p-values are in bold.

Supplementary figure 1. Flow chart of patient selection for the study

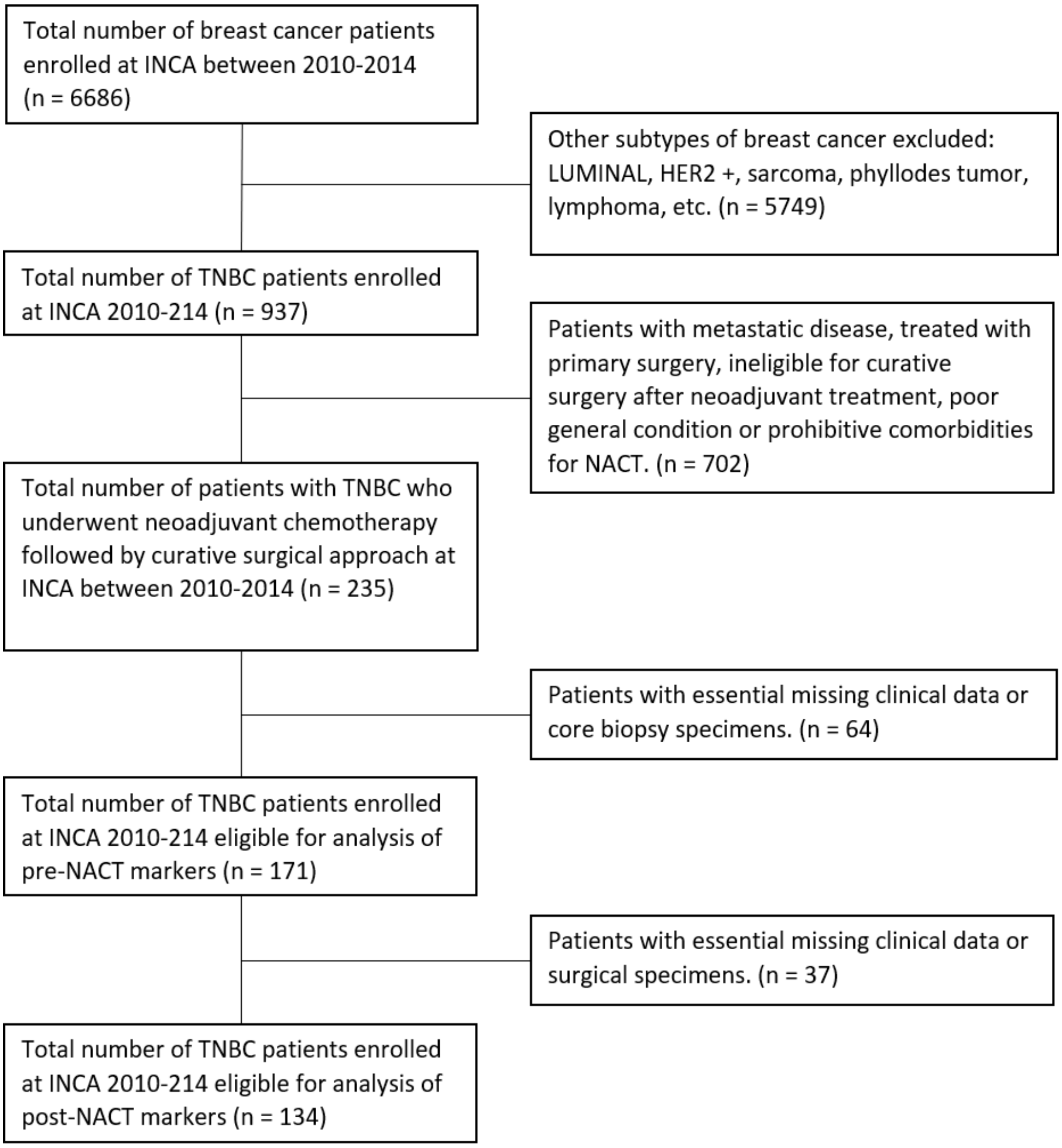

Supplement: Supplementary file 1 [file DataSheet_1.pdf]
